# Intra-tumor heterogeneity-resistant gene signature predicts prognosis and immune infiltration in breast cancer

**DOI:** 10.3389/fimmu.2025.1598858

**Published:** 2025-09-26

**Authors:** Haixing Shen, Qing Zheng, Jie Pan, Yukai Jin, Xiaohong Zheng, Qingyue Yuan, Da Tan, Qiang Zhou, Jingzhi Wang, Tianmiao Sun

**Affiliations:** ^1^ Affiliated Cixi Hospital, Wenzhou Medical University, Ningbo, Zhejiang, China; ^2^ Department of Gastrointestinal Surgery, The First Affiliated Hospital of Shantou University Medical College, Shantou, Guangdong, China; ^3^ Cixi Biomedical Research Institute, Wenzhou Medical University, Ningbo, Zhejiang, China; ^4^ Department of Neurosurgery, Shanghai Tenth People’s Hospital of Tongji University, Shanghai, China; ^5^ Department of Radiotherapy Oncology, The First People’s Hospital of Yancheng, Yancheng No.1 People’s Hospital, Affiliated Hospital of Medical School, Nanjing University, Yancheng, Jiangsu, China

**Keywords:** breast cancer, immunotherapy, immune infiltration, intra-tumor heterogeneity, prognosis, tumor microenvironment

## Abstract

**Background:**

Breast cancer (BC) remains a significant threat to human health, with substantial variations in prognosis and treatment responses. Intra-tumor heterogeneity (ITH) presents a critical challenge in developing reliable prognostic models.

**Methods:**

This study integrated multi-region RNA sequencing data from BC patients with the TCGA BC dataset. Genes resistant to sampling bias were identified by evaluating inter-patient heterogeneity (IPH) and ITH. A machine learning framework incorporating ten algorithms was used to construct a prognostic signature.The expression levels and oncogenic function of the prognostic genes were validated through RT-qPCR and in vitro experiments.

**Results:**

The signature, comprising CFL2 and SPNS2, demonstrated stable predictive performance in both training and validation cohorts (C-index > 0.6). High-risk patients exhibited enriched immune infiltration, particularly CD8+ T cells, and higher expression of immune checkpoint molecules, suggesting sensitivity to immunotherapy. A nomogram integrating risk score with clinical variables further improved prognostic accuracy. The dysregulation of signature genes was confirmed in BC cell lines.

**Conclusion:**

By minimizing ITH interference, this study developed a robust prognostic signature for BC, offering insights into the tumor immune microenvironment and potential therapeutic strategies.

## 1 Introduction

Breast cancer (BC) remained the most prevalent cancer among women globally in 2022, with its incidence rate continuing to rise. Notably, a more pronounced increase has been observed among younger women (1). Surgery is the cornerstone treatment for early-stage BC, particularly for managing primary tumors and regional lymph nodes. It is often combined with radiotherapy to enhance local control rates (2). In recent years, significant advancements have been made in BC treatment. These include molecularly targeted therapies, immune checkpoint inhibitors (ICIs), and novel antibody-drug conjugates (ADCs), which have led to marked improvements in patient survival rates (3, 4). Despite these advancements, some patients still respond poorly to current therapies, and there are considerable inter-individual variations in prognosis, ranging from several months to decades (5, 6). Accurate stratification of prognosis is crucial for monitoring disease progression and selecting appropriate treatment strategies. Currently, BC classification systems mainly include the American Joint Committee on Cancer (AJCC) TNM (tumor-node-metastasis) staging system and molecular subtypes (Luminal A, Luminal B, HER2-enriched, and triple-negative BC), which are widely implemented in clinical practice. While these assessment methods have proven useful, they exhibit various limitations in patient stratification and offer limited predictive accuracy. Furthermore, they fail to provide insights into the biological characteristics of BC that could explain clinical heterogeneity, highlighting the need for further improvements.

Over the past decades, breakthroughs in sequencing technology have significantly deepened our understanding of the molecular mechanisms underlying BC. Researchers have developed numerous gene-based prognostic models using tumor transcriptomic data, with the goal of guiding individualized treatment strategies (7, 8). However, these multi-gene signatures have yet to achieve widespread clinical translation. Challenges include the absence of standardized detection protocols and interference from intra-tumor heterogeneity (ITH) (9). Transcriptomic ITH poses a particular challenge, as it can lead to shifts in molecular subtyping and the heterogeneous distribution of therapeutic targets, ultimately compromising the reproducibility of biomarkers (10). Most existing prognostic models fail to account for the impact of ITH on feature stability, which may exacerbate prediction biases. Addressing this gap by integrating ITH into prognostic signature development is a critical step toward enhancing BC stratification precision and advancing personalized treatment.

Multi-region sequencing is a technique that involves sampling from different regions of the same tumor and performing high-throughput sequencing. This approach comprehensively captures molecular diversity and systematically quantifies the distribution patterns of ITH, thus improving the model’s ability to predict tumor evolution across regions (11). Multi-region sequencing helps mitigate the bias of single-point sampling, thus reducing the impact of tumor heterogeneity on prognostic models. Here, by integrating multi-region BC dataset and the TCGA (The Cancer Genome Atlas Program) BC dataset, we employed machine learning algorithms to identify prognosis-related genes resistant to sampling bias and constructed a risk signature. This signature was validated in an external independent cohort, demonstrating robust survival prediction performance. The signature was also proven to be a biologically specific marker associated with the tumor microenvironment (TME) and capable of predicting responses to immunotherapy. Furthermore, we combined the signature with clinical variables to create a dynamic nomogram for individualized risk assessment.

## 2 Methods

### 2.1 Data preparation and processing

We first downloaded sequencing data from TCGA breast invasive carcinoma (TCGA-BRCA) dataset (n=1231), including 1,118 tumor samples and 113 normal samples. We excluded cases with missing survival time or endpoint event status. To minimize interference from non-cancer-related deaths, we excluded patients with survival times less than 30 days. The expression data were normalized using DESeq2 (Version 1.44) and transformed with VST, serving as the training set to construct the prognostic model. Additionally, we incorporated multi-region bulk-RNA data from BC patients (32 samples from 10 patients) provided by Aneja et al. to explore markers less affected by ITH (12). The GSE42568 dataset (104 BC cases) was downloaded from the GEO database for validating the prognostic signature. “Masked Somatic Mutation” data was selected as the somatic mutation dataset for TCGA-BRCA.

### 2.2 Immune infiltration analysis

ESTIMATE (Estimation of STromal and Immune cells in MAlignant Tumours using Expression data) (Version 1.0.13) was used to infer the proportions of stromal and immune cells in tumor samples (13). CIBERSORT (Cell-type Identification By Estimating Relative Subsets Of RNA Transcripts), a deconvolution algorithm-based tool, was employed to quantify the relative proportions of 22 immune cell subtypes within the TME (14). quanTIseq was used to quantify the absolute abundance of immune cells (15). The IOBR (Immuno-Oncology Biological Research) (Version 0.99.8) was utilized to analyze metabolic features in the TME, identifying key characteristics associated with the survival of TCGA-BRCA patients (16). The Immunophenoscore (IPS) was used to reflect the immune activity of the TME, aiding in evaluating patients’ potential response to ICIs (17).

### 2.3 Quantification of gene heterogeneity

Gene expression heterogeneity was assessed by evaluating inter-patient heterogeneity (IPH) and ITH (10). These two sets of scores were used to measure the variability in expression between different patients and within different regions of the same tumor, quantified using standard deviation. For ITH, the standard deviation of expression within the group of samples was calculated for each gene individually. For IPH, one sample was randomly selected from each region to construct new cross-group sample subsets, generating a total of 10 independent resampling iterations. For each resampled subset, the measure of cross-group gene expression variability was calculated.

### 2.4 Functional enrichment analysis

Kyoto Encyclopedia of Genes and Genomes (KEGG) was utilized for gene functional annotation and enrichment analysis to identify biological pathways or metabolic processes in which the genes are involved.

### 2.5 Construction of prognostic signature

Reducing ITH helps maintain the stability of prognostic markers. Additionally, enhancing IPH facilitates the identification of unique prognostic markers for different individuals, enhances the discriminative ability of prognostic models, and strengthens patient stratification for tailored treatment strategies. First, we divided genes from multi-region samples into groups based on the mean standard deviation levels, categorizing them as exhibiting high/low interpatient heterogeneity (IPH) and high/low ITH. Genes displaying both high IPH and low ITH were subjected to intersection analysis with differentially expressed genes (DEGs) from the TCGA-BRCA dataset. Using a threshold of 0.05, these genes underwent further screening via univariate Cox regression and proportional hazards (PH) assumption testing. Subsequently, the selected genes were processed through an integrated machine learning framework that incorporates ten classical algorithms (Mime1, Version 0.0.0.9000): random survival forest (RSF), elastic net (Enet), stepwise Cox regression (StepCox), CoxBoost, partial least squares regression for Cox models (plsRcox), supervised principal component analysis (superPC), generalized boosted regression modeling (GBM), survival support vector machine (survival-SVM), ridge regression, and least absolute shrinkage and selection operator (Lasso) (18). This approach generated 117 algorithm combinations, which were then trained and evaluated on the TCGA training dataset using K-fold cross-validation. The risk score is calculated using the following formula:

Risk score = Σ (Expi × Coefi).

Expi and Coefi represent the expression levels and coefficients of prognostic genes.

### 2.6 Nomogram construction

Following the derivation of risk scores, we further integrated clinicopathological variables to construct an individualized prognostic prediction model. First, univariate Cox proportional hazards regression analysis was performed to assess the impact of each variable (including risk group, tumor stage, age, and sex) on overall survival. Variables reaching the significance threshold (P < 0.05) in univariate analysis were subjected to multivariate Cox regression to identify independent prognostic predictors. Based on the multivariate Cox regression results, a nomogram was constructed using the rms package (Version 6.8-0) in R. In the nomogram, each variable was assigned points proportional to its regression coefficient. The total score for each patient, calculated by summing the points across all variables, was mapped to a scale for predicting survival probabilities or risk rates.

### 2.7 Drug sensitivity analysis

Using the TCGA-BRCA gene expression profiles and the cgp2016ExprRma dataset, we employed the R package pRRophetic (Version 0.5) to predict the half-maximal inhibitory concentration (IC50) values for each drug, representing tumor cell sensitivity. To explore the relationship between the risk score and drug sensitivity, we performed a Spearman rank correlation test to calculate the correlation coefficient between the risk score and the IC50 of each drug. Candidate drugs with an absolute correlation value exceeding 0.3 were retained.

### 2.8 Cell culture

Human normal breast cell lines (MCF-10A) and BC cells (MDA-MB-231, MCF-7, BT-474, SKBR3) were obtained from ATCC. Cells were maintained at 37°C under 5% CO_2_ atmosphere.

### 2.9 RT-qPCR analysis

Total RNA was extracted from the cells using TRIzol reagent (Invitrogen, USA). Subsequently, reverse transcription was performed using the PrimeScript RT Reagent Kit acquired from TaKaRa. The following PCR conditions were employed on the StepOnePlus PCR System (TaKaRa) using 2× RealStar Power SYBR Mixture (TaKaRa): an initial predenaturation at 95°C for 2 min, then 95°C for 15 s, 60°C for 30 s, and 72°C for 30 s, for a total of 40 cycles. The PCR amplification primer sequences were as follows: CFL2, forward: 5’- AGCCGAGGGCACTATGGTAA-3’, reverse: 5’-AGAAGCCTTGGAGGCCAAAA-3’; SPNS2, forward: 5’- GACAGGTACACCGTGGCAG-3’, reverse: 5’- CCAGGTAGCCGAAGATGGG-3’; β-actin, forward: 5′-TCCATCATGAAGTGTGACGT-3’, reverse: 5’-GAGCAATGATCTTGATCTTCAT-3′. Relative mRNA expression levels were calculated using the comparative Ct (2−ΔΔCt) method and normalized to β-actin as the endogenous control. Experiments included three independent biological replicates.

### 2.10 Cell function assays

The short hairpin (sh)RNAs for CFL2 knockdown (sh-CFL2), CFL2 overexpression (oe-CFL2), SPNS2 knockdown (sh-SPNS2), SPNS2 overexpression (oe-SPNS2), and negative controls (sh-NC and oe-NC) plasmids were obtained from GenePharma (China). Cells were seeded in 24-well plates at a density of 3×10^4^ cells/well. When cell confluence reached approximately 70%, transfection was performed using Lipofectamine 3000 (Invitrogen, USA). After 48 hours of transfection, cells were collected for experimental studies.

Transfected MDA-MB-231 cells were seeded into 96-well plates at a density of 2,500 cells/well. At 24, 72, and 120 hours post-seeding, 10 μL of CCK-8 reagent was added to each well, followed by incubation for 2 hours. The optical density (OD) at 450 nm was measured using a spectrophotometer. Growth curves were generated, and cell viability was calculated for each group.

Transfected MDA-MB-231 cells were plated in six-well plates and incubated for 24 hours in a culture incubator. After colony formation, cells were fixed with 4% paraformaldehyde for 30 minutes, stained with 0.1% crystal violet for 20 minutes, air-dried, photographed, and images were recorded.

Transfected MDA-MB-231 cells were trypsinized and resuspended in serum-free medium. For the invasion assay, cell suspensions were placed in the upper chamber, while the lower chamber was filled with 600 μL of medium containing 20% fetal bovine serum. The upper chamber was coated with Matrigel and allowed to solidify at 37°C for 2 hours. Subsequently, cell suspensions were added, and the assay proceeded for 24 hours. After incubation, chambers were washed with PBS at room temperature, fixed with 4% paraformaldehyde for 30 minutes, and stained with 0.1% crystal violet for 20 minutes. After drying, images were captured and saved for analysis using an inverted microscope at room temperature.

### 2.11 Statistical methods

For normally distributed data with equal variances, the t-test was used to compare mean differences between two independent or paired samples. For non-normally distributed data, the Mann-Whitney U test was applied to assess group differences. The Spearman rank correlation coefficient was used to evaluate the relationship between variables. Time-dependent receiver operating characteristic (ROC) curves were generated to evaluate the model’s predictive accuracy at different time points. Calibration curves were plotted using the Bootstrap resampling method (1,000 iterations) to assess the agreement between model-predicted survival probabilities and observed survival rates. A curve close to the diagonal (ideal fit) indicated good model calibration. Harrell’s concordance index (C-index) was calculated to quantify the model’s global predictive ability for survival outcomes. Univariate and multivariate Cox regression analyses were performed to identify prognostic factors. The proportional hazards assumption was tested using Schoenfeld residuals. A p-value < 0.05 was considered statistically significant.

## 3 Results

### 3.1 IPH and ITH in BC

**Figure 1:**
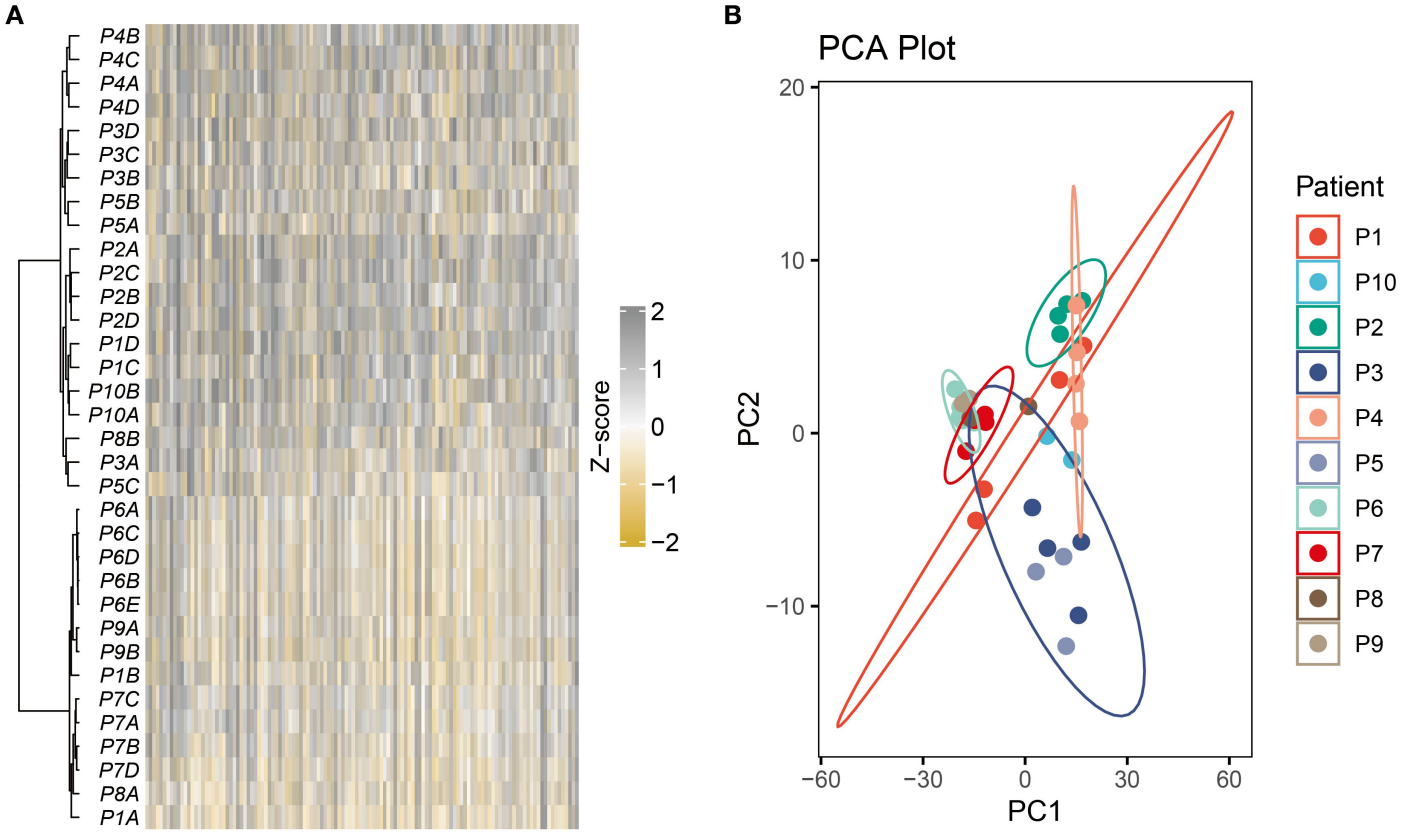
Strong IPH was observed in the multi-region BC cohort. **(A)** The heatmap displayed unsupervised hierarchical clustering of BC samples in the multi-region BC cohort based on the top 500 variable genes. **(B)** Principal component analysis of the entire transcriptome profile of the multi-region BC cohort.

Unsupervised hierarchical clustering was performed on the most variable genes across 32 multi-regional primary BC tissues from 10 patients. Results showed that almost all patients exhibited distinct clustering patterns, with regions from the same tumor consistently grouped together (**Figure 1A**). This revealed strong IPH and highlighted consistent ITH profiles. Dimensionality reduction of the entire transcriptome profiles further confirmed that regions from the same tumor primarily clustered by patient (**Figure 1B**).

**Figure 2:**
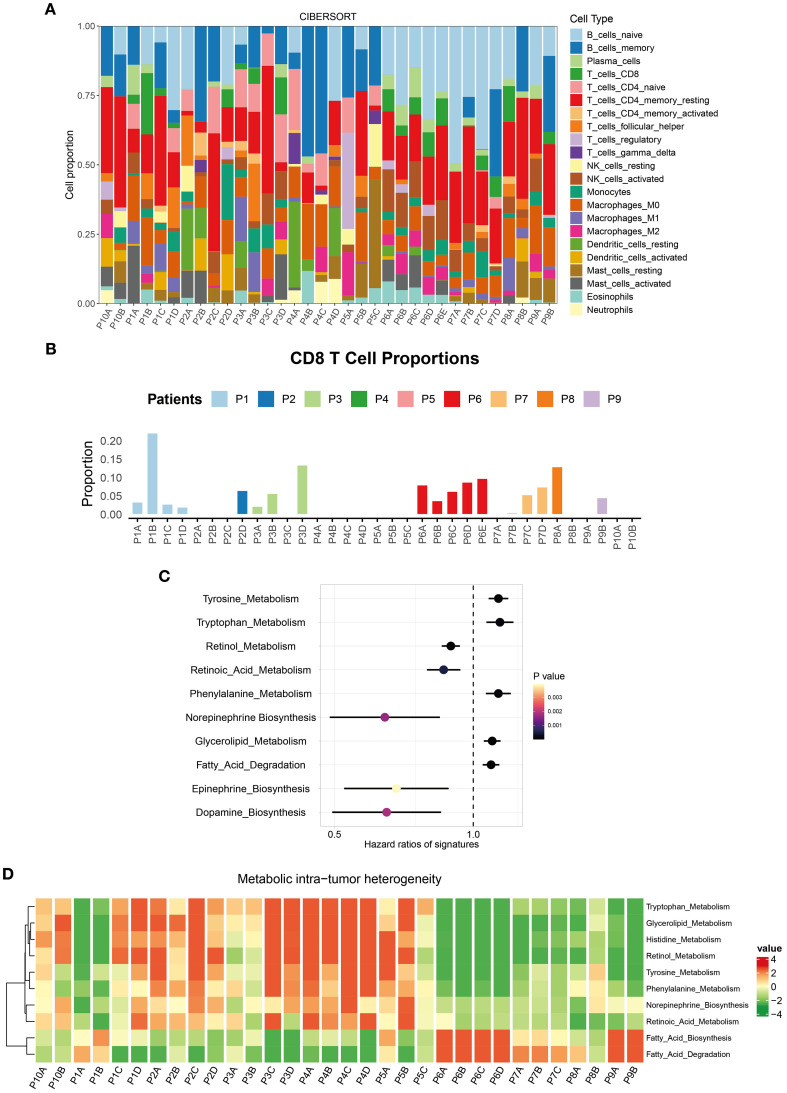
Immune microenvironment ITH in the multi-region BC cohort. **(A)** Stacked percentage plot illustrated the proportional composition of cell subtypes across different regions. **(B)** Bar chart showed the infiltration abundance of CD8+ T cells in different regions. **(C)** IOBR identified the ten TME metabolic signatures with the most significant impact on BC patient prognosis. **(D)** Heatmap displayed the enrichment levels of metabolic signatures across different regions.

Using the CIBERSORT algorithm, we quantified the proportions of 22 immune cell types across tumor regions. Significant differences in immune cell composition were observed, even within the same tumor (**Figure 2A**). For instance, the P1B region exhibited CD8+ T cell infiltration exceeding 20%, indicating strong immune activity and a potential robust anti-tumor response. In contrast, other regions of the same tumor showed CD8+ T cell proportions below 5%, suggesting weaker immune activity (**Figure 2B**).

By employing the IOBR tool, we identified 10 metabolic signatures closely associated with BC patient survival (**Figure 2C**). These scores encompassed core metabolic pathways within the TME. Heatmaps revealed metabolic heterogeneity across different regions of the same tumor. Tryptophan metabolism was significantly upregulated in the P1C and P1D regions but downregulated in P1A and P1B (**Figure 2D**). This metabolic pathway plays diverse mechanistic roles in the TME: Its degradation product kynurenine promotes immune escape by inhibiting effector T cell function (19); enhanced activity may reflect competitive nutrient uptake by tumor cells, altering the metabolic balance of the local microenvironment (20). Such ITH in the metabolism underscores the extreme complexity of the TME, influencing both tumor cell behavior and immune cell function.

In exploring genomic heterogeneity in breast cancer, we focused on Mutant-Allele Tumor Heterogeneity (MATH). This metric quantifies ITH by calculating the dispersion of mutant allele frequencies in tumor samples. A higher MATH value suggests greater genetic diversity among tumor subclones. Based on the MATH values computed for the TCGA-BRCA cohort samples, we further analyzed its relationship with clinical variables. The results showed that there was no significant clinical correlation between MATH values and factors such as age, treatment regimens, overall survival, or most TNM stages (**Supplementary Figures S1A, B**). However, a statistically significant difference was observed in MATH between Stage I and Stage II tumors (**Supplementary Figure S1A**). This finding suggests that genomic heterogeneity may increase as tumors progress from early-stage to slightly more advanced Stage II. In contrast, late-stage tumors (Stage III and IV) may generally reach a high baseline level of heterogeneity, with dominant clones expanding significantly within these tumors. This could be a reason why no significant differences in MATH values were observed between different late-stage subgroups.

### 3.2 Quantification of gene heterogeneity

**Figure 3:**
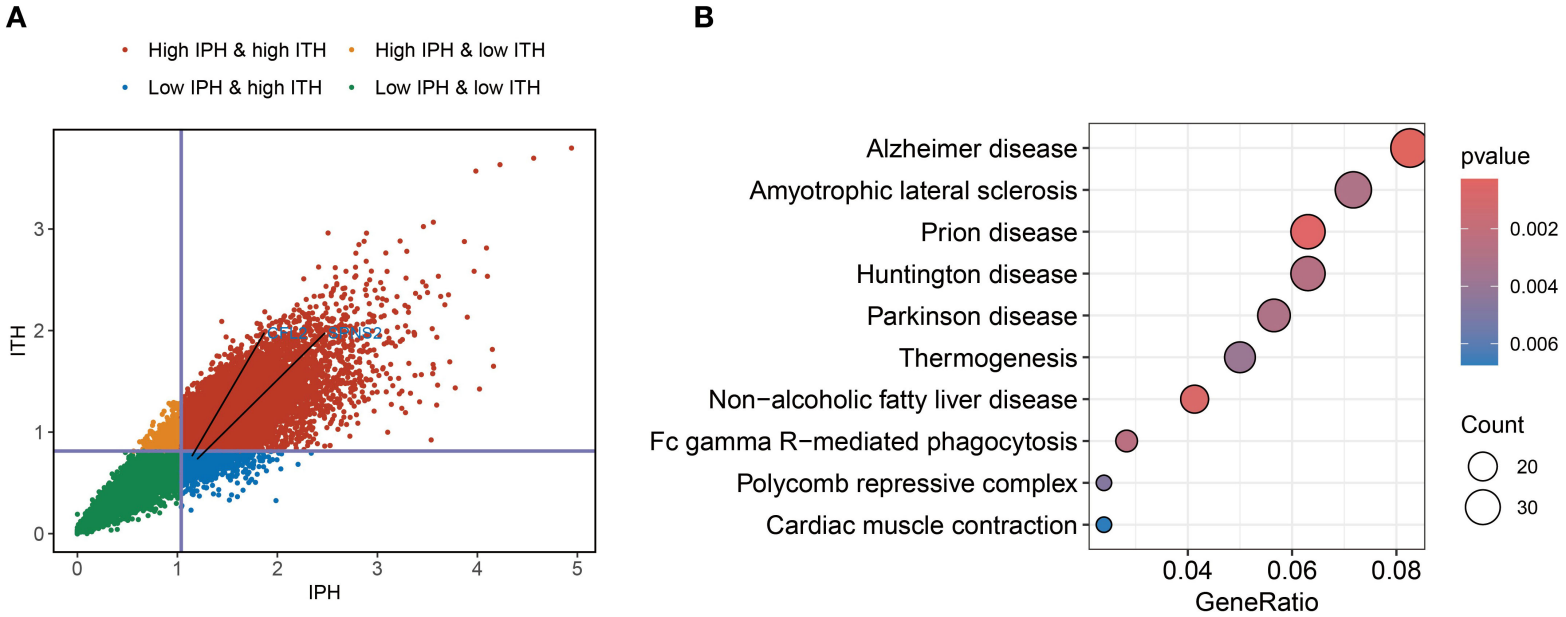
Identification of ITH-resistant genes. **(A)** Quadrant plot of gene expression variability calculated using the multi-region BC cohort. The plot was divided into four quadrants based on the average inter-patient (vertical line) and intra-tumor (horizontal line) heterogeneity scores. **(B)** KEGG enrichment analysis of ITH-resistant genes.

To define RNA heterogeneity, we derived IPH and ITH metrics for each gene using multi-regional BC samples. These metrics were categorized into high or low groups based on their mean values, resulting in four RNA heterogeneity quadrants for BC. Genes with high IPH and low ITH (Blue points in **Figure 3A**) exhibit notable variability across patients but high homogeneity within tumors. This characteristic not only helps minimize biases during sample collection but also offers potential molecular markers for precise patient stratification.

To further explore the biological functions of genes with high IPH and low ITH, we conducted pathway enrichment analysis. The results revealed significant enrichment of genes related to FcγR-mediated phagocytosis. Fcγ receptors, as critical components of the immune system, play a key role in antibody-dependent cellular cytotoxicity (7). Additionally, we observed significant enrichment of genes associated with thermogenesis (**Figure 3B**). This finding suggests that energy metabolism pathways may play a more complex regulatory role in the progression of BC.

### 3.3 Construction and validation of prognostic signature

**Figure 4:**
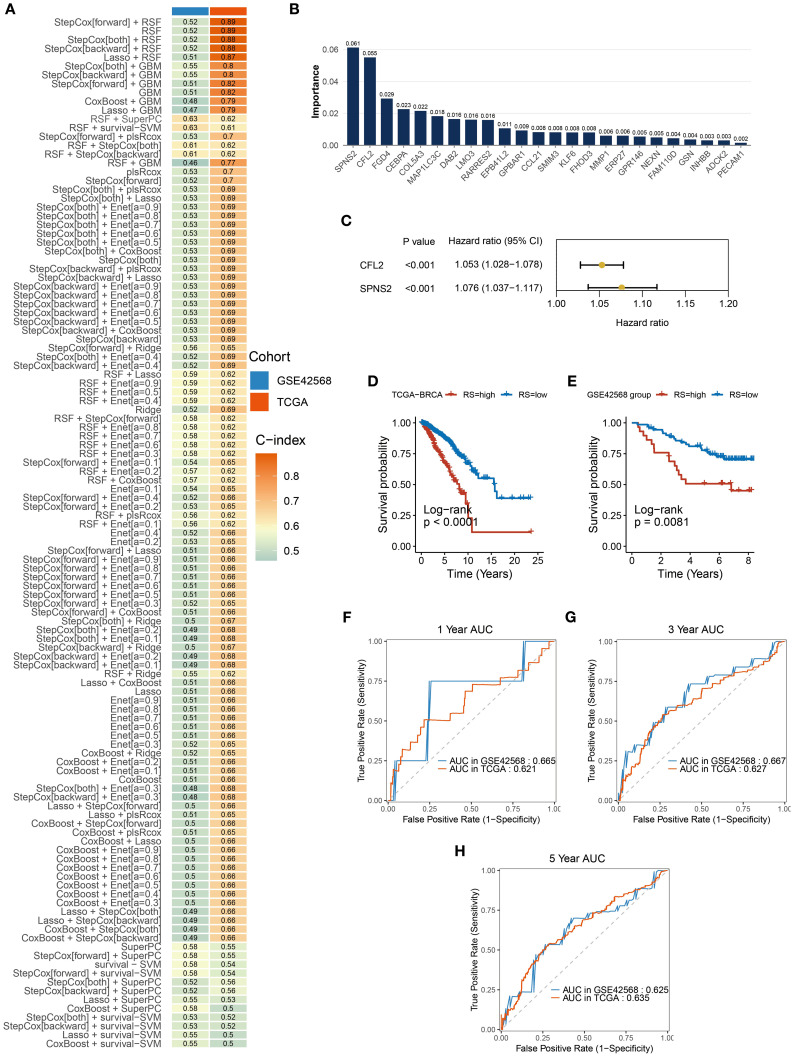
Construction and validation of prognostic signatures. **(A)** A total of 117 predictive models were built using a machine learning framework, and the C-index of each model was further calculated. **(B)** Importance of the 24 genes contributing to survival time in the random survival forest model. **(C)** StepCox-both identified CFL2 and SPNS2. **(D, E)** Kaplan-Meier analysis showed significant prognostic differences between high-risk and low-risk groups in TCGA-BRCA and GSE42566 datasets. **(F–H)** The signature’s AUC for 1-year, 3-year, and 5-year predictions in TCGA-BRCA and GSE42566 datasets.

Analysis of the intersection between DEGs from the TCGA-BRCA dataset and high-IPH/low-ITH genes identified differentially expressed ITH-resistant genes. To assess their prognostic value, univariate Cox regression analysis and PH assumption testing were conducted, yielding 24 prognostic candidate genes. Within a comprehensive machine learning framework (**Figure 4A**) incorporating 10 classical algorithms and 117 diverse algorithm combinations, the RSF and StepCox-both algorithms were selected based on C-index results to establish a BC prognostic risk assessment model. Initially, the random survival forest quantified the importance of the 24 genes (**Figure 4B**). Further, high-conserved variable selection via minimal depth filtering identified three genes (CFL2, FGD4, and SPNS2). After applying StepCox-both, the final model retained CFL2 and SPNS2 (**Figure 4C**). The prognostic risk signature demonstrated stable predictive performance in both the TCGA-BRCA cohort and GSE42568 validation set, with C-index values of 0.61 and 0.62, respectively (**Figure 4A**). BC samples were stratified into high- and low-risk groups based on median risk scores. Risk stratification analysis revealed significant prognostic divergence between groups (**Figures 4D, E**). High-risk patients exhibited poorer clinical outcomes in both TCGA-BRCA and GSE42568 datasets, confirming the signature’s clinical validity. Time-dependent ROC analysis showed robust performance in TCGA-BRCA, with 1-year, 3-year, and 5-year AUCs of 0.621, 0.627, and 0.635 (**Figures 4F–H**). In GSE42566, while short-term prediction improved (1-year and 3-year AUCs: 0.665 and 0.667), 5-year performance declined to 0.625 (**Figures 4F–H**).

### 3.4 Construction of an individualized prognostic nomogram

**Figure 5:**
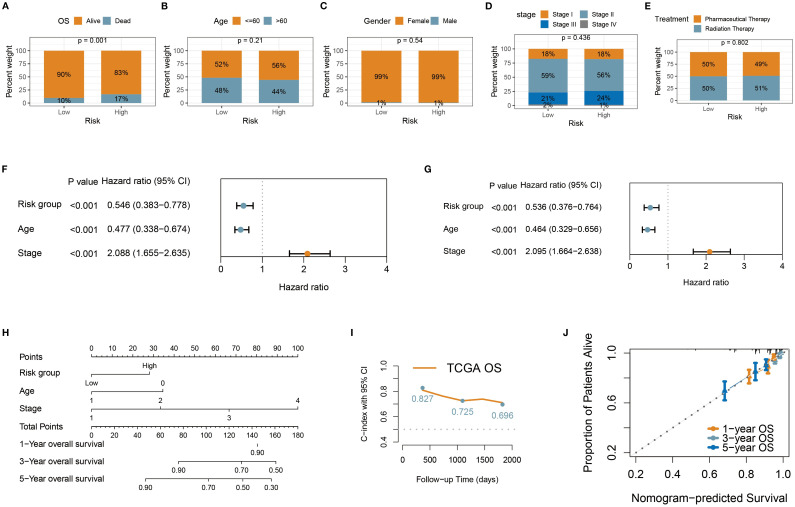
Construction of personalized prognostic nomogram. **(A–E)** Stacked percentage plots illustrated the proportional composition of survival status, age, gender, treatment status, and tumor stage in the high-risk and low-risk groups. **(F, G)** Univariate and multivariate Cox regression analyses were used to assess the impact of risk group and clinical variables on patient survival time. **(H)** The nomogram enables clinicians to quickly evaluate patient prognosis. **(I)** The nomogram demonstrated robust discriminatory ability, with the C-index consistently close to 0.7. **(J)** Calibration curves showed high agreement between predicted and actual outcomes, further validating its accuracy.

Through chi-square tests on the characteristics of TCGA BC patients in the high-risk and low-risk groups, we identified some noteworthy clinical feature distribution patterns. Mortality cases were significantly higher in the high-risk group (17%) compared to the low-risk group (10%) (p < 0.05) (**Figure 5A**). This validated the discriminant ability of the prognostic risk signature. However, comparisons of other clinicopathological features (age, gender, treatment status, and tumor stage) showed no significant differences between the two groups (p > 0.05) (**Figures 5B–E**), which suggests that the prognostic risk signature may be based on different biological foundations than traditional clinical indicators, providing additional prognostic information. Moreover, the consistency in feature distribution indicates that the risk score may be independent of conventional clinical factors, offering a new dimension for prognosis assessment. Thus, we conducted a systematic prognostic factor analysis. First, in the univariate Cox analysis, variables with a significance threshold of P < 0.05 were screened, revealing that age, tumor stage, and risk group met the criteria as significant predictors (**Figure 5F**). In multivariate Cox regression analysis, these variables were confirmed as independent prognostic factors (P < 0.05), reaffirming the importance of traditional indicators while validating the independent prognostic value of the risk score (**Figure 5G**). To visualize these results, we constructed a nomogram using the rms package in R. The nomogram transforms multivariate Cox regression results into a scoring system, enabling clinicians to quickly assess patient prognosis (**Figure 5H**). The model demonstrated good discriminatory ability, with a C-index consistently nearly 0.7 (**Figure 5I**), and calibration curves showed strong agreement between predicted and actual outcomes, further validating its accuracy (**Figure 5J**).

### 3.5 Immune infiltration and immunotherapy response of prognostic signature

**Figure 6:**
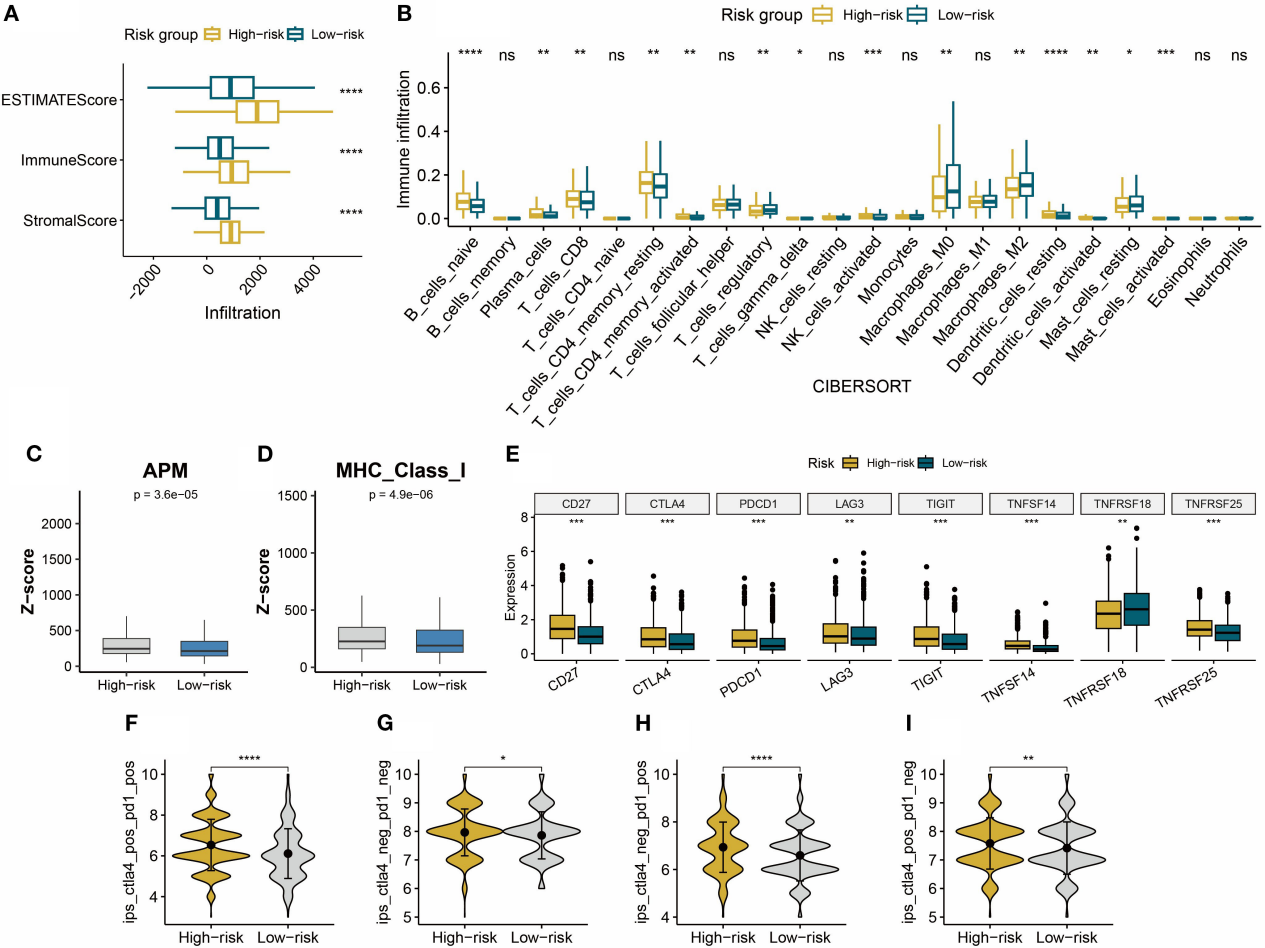
**(A)** The ESTIMATE algorithm quantified the Stromal Score, Immune Score, and ESTIMATE Score in high- and low-risk BC groups. **(B)** CIBERSORT quantified the relative proportions of 22 immune cell subtypes in high- and low-risk BC groups. **(C, D)** APM and MHC-I calculated based on z-scores in high- and low-risk groups. **(E)** Immune checkpoint expression status in high- and low-risk groups. **(F–I)** Compared to the low-risk group, the IPS score is significantly upregulated in the high-risk group. *P < 0.05, **P < 0.01, ***P < 0.001, ****P < 0.0001, ns: not significant.

Through the analysis of the immune microenvironment, we uncovered a complex landscape of immune infiltration in high- and low-risk groups. Using the ESTIMATE algorithm, we evaluated the overall immune infiltration levels and found that the high-risk group exhibited more pronounced immune infiltration characteristics (**Figure 6A**). Employing the CIBERSORT algorithm, we performed detailed immune cell subset analysis. A significant elevation of CD8+ T cells was observed in the high-risk group, indicative of an active immune state in these patients (**Figure 6B**). Notably, CIBERSORT analysis revealed reduced proportions of regulatory T cells and M2 macrophages in the high-risk group (**Figure 6B**). We also used quanTIseq to quantify the absolute abundance of immune cells in the TME (**Supplementary Figure S2**). Further analysis revealed that the z-scores related to MHC-I molecules and the antigen processing machinery (APM) were significantly elevated in the high-risk group (**Figures 6C, D**). The upregulation of MHC-I and APM indicates stronger antigen-presenting capabilities in immune cells, which may contribute to the increased infiltration of CD8+ T cells. In the high-risk group, the expression levels of several key immune checkpoint molecules (including CD27, CTLA4, PDCD1 (PD-1), LAG3, TIGIT, TNFSF14, TNFRSF25) were significantly upregulated. This suggests more active but suppressed tumor-immune interactions in high-risk patients and provides critical insights for selecting appropriate immunotherapy targets (**Figure 6E**). This study further explored the correlation between the prognostic risk signature and the prediction of immunotherapy response. It was found that the IPS was significantly higher in the high-risk group compared to the low-risk group. Since the IPS is closely associated with immunotherapy responsiveness, the results suggest that high-risk patients may exhibit greater sensitivity to immunotherapy (**Figures 6F–I**).

### 3.6 Mutational landscape of risk subgroups

**Figure 7:**
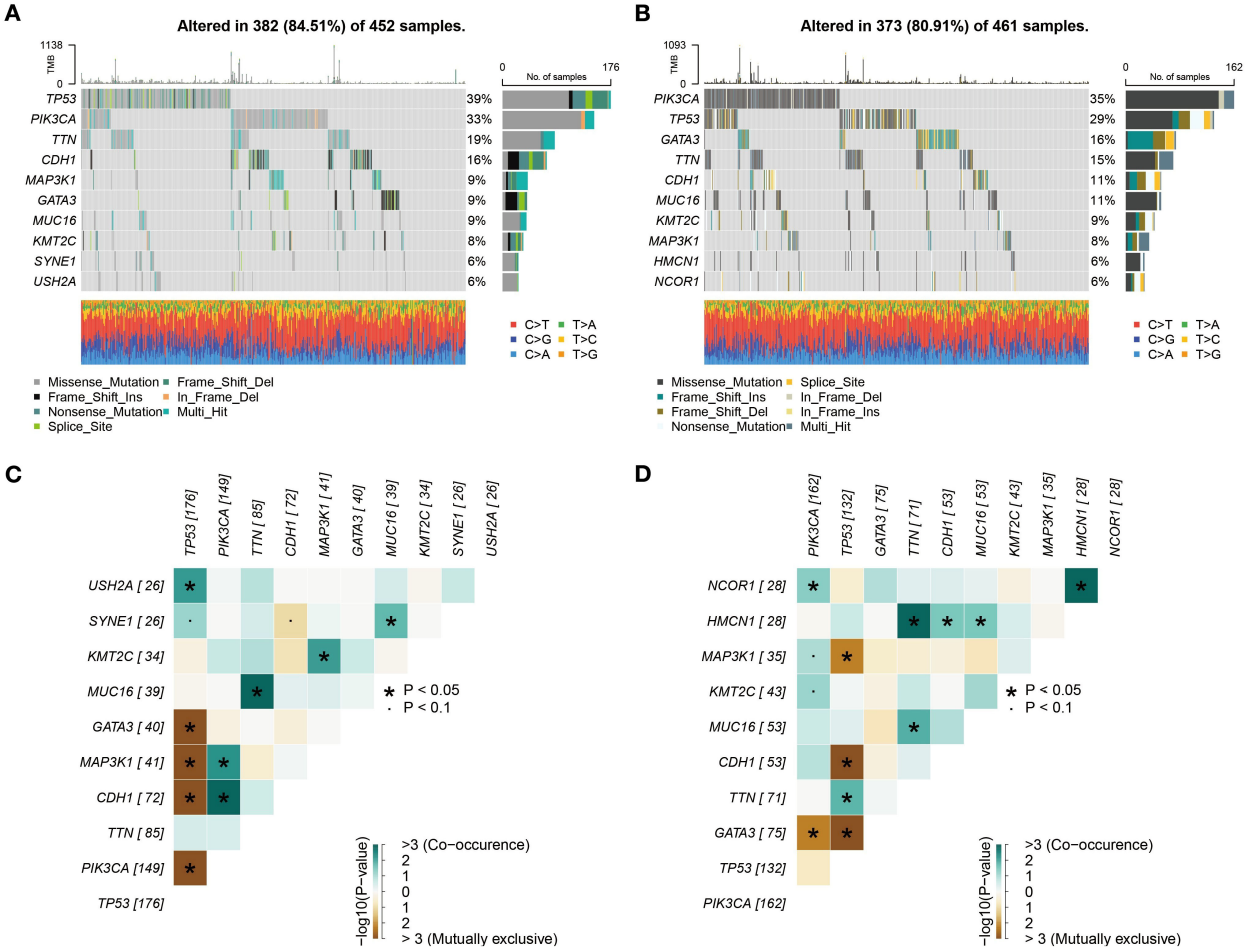
Subgroup mutation analysis. **(A, B)** Comprehensive analysis based on whole exome sequencing data revealed that the mutation rates of TP53 and PIK3CA in both high- and low-risk groups exceed 25%. **(C, D)** Analysis of mutation co-occurrence/exclusion in high- and low-risk groups.

The waterfall plot revealed that TP53 and PIK3CA were the most frequently mutated genes in both the high- and low-risk groups (**Figures 7A, B**). This finding aligns with previous findings on BC, confirming the central role of these genes in BC development (21). As a key tumor suppressor gene, mutations in TP53 may lead to genomic instability and pro-tumorigenic properties, which could partially explain the more aggressive features observed in the high-risk group. On the other hand, activating mutations in PIK3CA likely play a significant role in promoting cell proliferation and survival through dysregulation of the PI3K/AKT pathway (21). Although slight differences in the mutation frequencies of TP53 and PIK3CA were observed between the two groups, overall, they exhibited remarkable convergence in the types and frequencies of the most common mutated genes. This similarity suggests that differences in high-frequency mutations alone may not sufficiently explain the significant clinical prognostic disparities between the two groups.

To further explore the interaction patterns among gene mutations, we constructed a heatmap to illustrate the co-occurrence and mutual exclusivity relationships of the top 10 mutated genes. The analysis revealed that the high- and low-risk groups exhibited similar patterns in mutation associations, both in terms of the frequency and strength of co-occurrence or mutual exclusivity for specific genes (**Figures 7C, D**). From a biological perspective, this similarity indicates that the core molecular pathways influencing BC development may be conserved between the high- and low-risk groups. Despite their significant differences in prognosis, both groups may rely on similar molecular mechanisms driving tumor progression.

### 3.7 Drug sensitivity analysis

**Figure 8:**
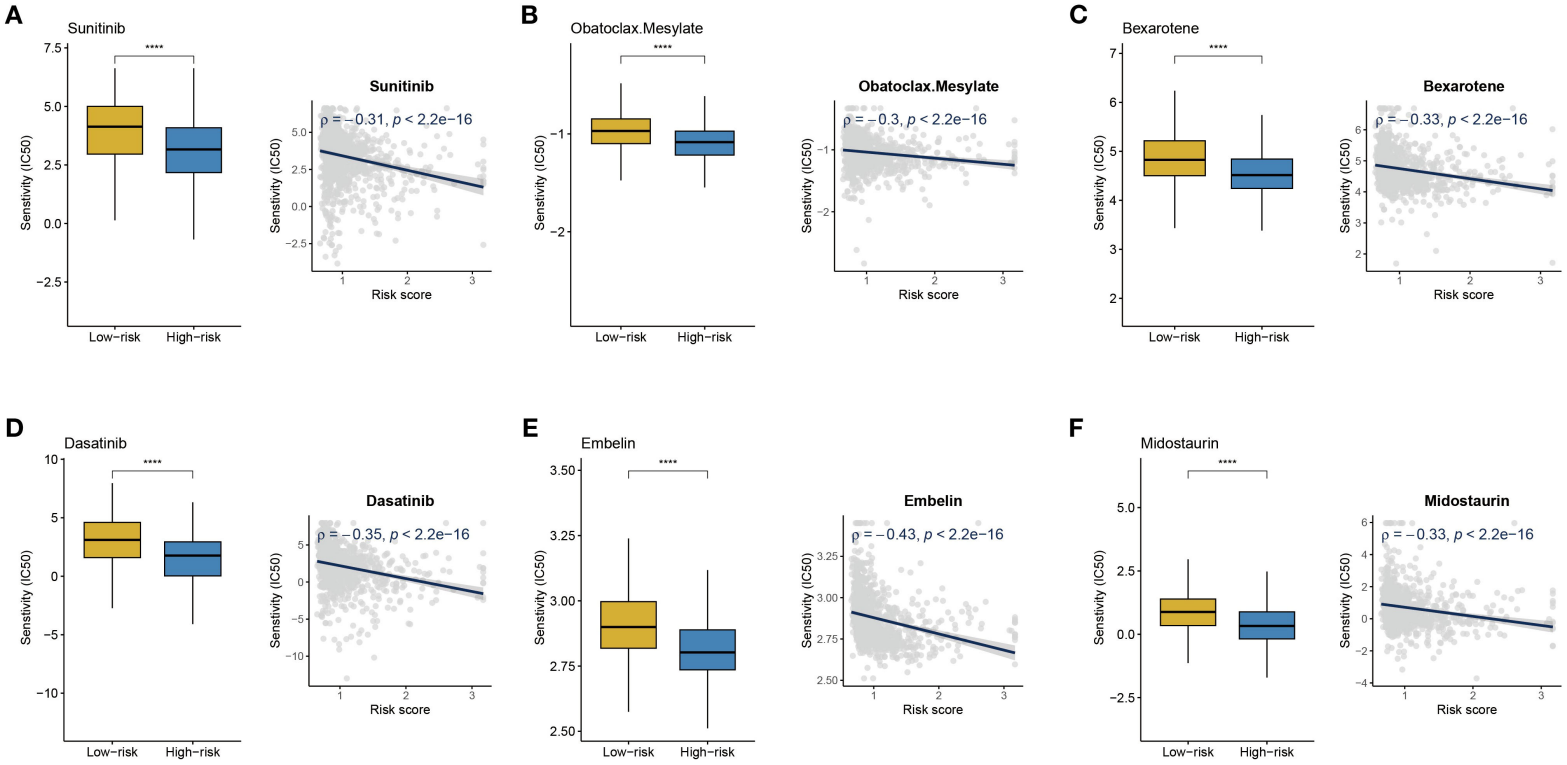
Drug sensitivity analysis. **(A-F)** IC50 values of Sunitinib, Obatoclax Mesylate, Midostaurin, Embelin, Dasatinib, and Bexarotene in high- and low-risk BC groups; correlation analysis between risk score and IC50 of different drugs. ****P < 0.0001.

To identify potential therapeutic drugs that are more effective for high-risk BC patients, this study conducted a drug sensitivity prediction analysis based on gene expression profile data using the R package pRRophetic. A group of potential therapeutic drugs for high-risk BC patients was identified, including Sunitinib, Obatoclax Mesylate, Midostaurin, Embelin, Dasatinib, and Bexarotene (**Figures 8A–F**). Previous studies support the potential use of these drugs for the treatment of BC patients. Sunitinib, as a multi-target tyrosine kinase inhibitor, targets the VEGFR and PDGFR pathways, which may be related to angiogenesis and aggressiveness in high-risk BC (22). In HR+/HER2- BC, a synergistic effect between anti-angiogenesis and hormonal therapy has been observed (23, 24). Dasatinib, as an SRC family kinase inhibitor, might exert therapeutic effects by inhibiting active tumor-stroma interactions in high-risk BC (25). In HER2-positive BC, Dasatinib combined with Neratinib significantly suppresses cell proliferation and induces stronger apoptosis and migration inhibition. This synergy is particularly evident in trastuzumab-resistant or Neratinib-acquired resistance models (26). Bexarotene, a selective retinoid X receptor agonist, activates RXR receptors to regulate the transcription of tumor-related genes, influencing cell proliferation, differentiation, and apoptosis pathways (27, 28). Obatoclax Mesylate and Midostaurin, as BCL-2 family protein and PKC inhibitors, may target the apoptosis-resistant mechanisms unique to high-risk BC (29, 30). Embelin exhibits stronger growth inhibition against TNBC tumors enriched with α-SMA-expressing cancer-associated fibroblasts (CAFs), such as the 4T1 model, and reduces the expression of pro-fibrotic markers like PDGFRA (31).

### 3.8 CFL2 and SPNS2 were upregulated in BC

**Figure 9:**
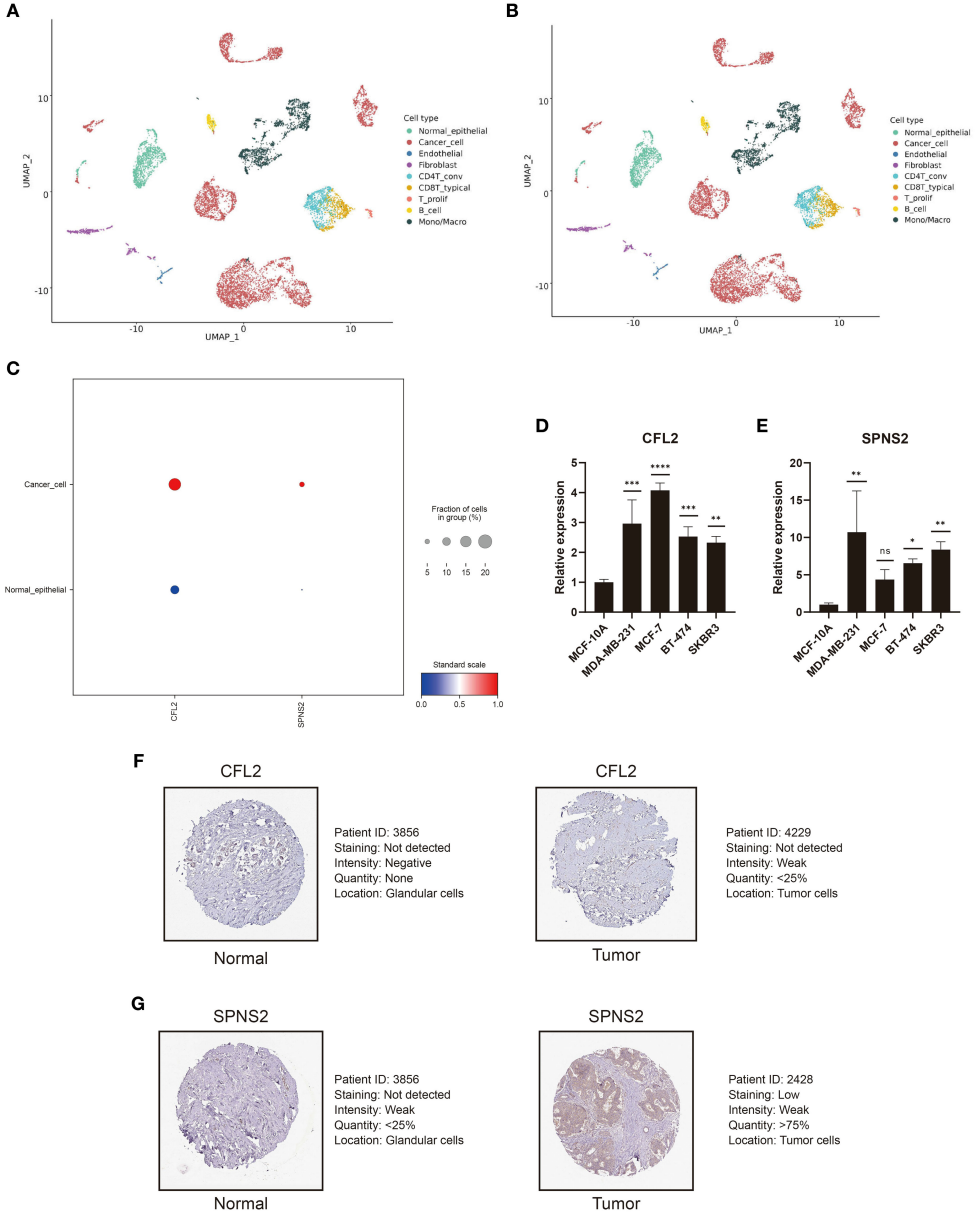
CFL2 and SPNS2 are upregulated in BC. **(A)** UMAP dimensionality reduction analysis of GSE148673, dividing the single-cell data into 9 cell clusters. **(B)** Proportion and quantity distribution of each cell type in GSE148673. **(C)** Compared to normal epithelial cells, CFL2 and SPNS2 are upregulated in cancer cells. **(D, E)** Compared to normal breast epithelial cells, RT-qPCR showed that CFL2 and SPNS2 are upregulated in BC cell lines. **(F, G)** Representative images of CLF2 and SPNS2 from the HPA database. *P < 0.05, **P < 0.01, ***P < 0.001, ****P < 0.0001, ns: not significant.

Based on the BC single-cell sequencing dataset GSE148673, we employed the scCancerExplorer analytical platform for cell clustering and expression profiling (32). Through UMAP-based dimensionality reduction, single-cell data were categorized into nine distinct cellular clusters (**Figure 9A**). Cancer cells (red) and normal epithelial cells (green) were completely segregated, indicating pronounced transcriptional heterogeneity between the two. Cancer cells accounted for 52.6%, dominating the cellular composition, which suggests high tumor purity (**Figure 9B**). Importantly, we revealed that CFL2 and SPNS2 exhibit upregulated average expression in cancer cells compared to normal epithelial cells (**Figure 9C**). To examine the expression of CFL2 and SPNS2, RT-qPCR was conducted in BC cell lines. Results revealed that CFL2 was significantly upregulated in MDA-MB-231, MCF-7, BT-474, and SKBR3 cells compared with normal mammary epithelial cells (MCF-10A) (**Figure 9D**). Additionally, SPNS2 exhibited significant upregulation in MDA-MB-231, BT-474 and SKBR3. Although upregulated in MCF-7, no statistical significance was observed (**Figure 9E**). To analyze the expression of CFL2 and SPNS2 at the protein level, we downloaded immunohistochemical images of breast tumor tissues and normal tissues from the HPA. The differential protein expression between BC and normal tissues was consistent with our transcriptomic analysis results (**Figures 9F, G**).

### 3.9 Knockdown of CFL2 or SPNS2 inhibits the proliferation and invasion of BC cells

**Figure 10:**
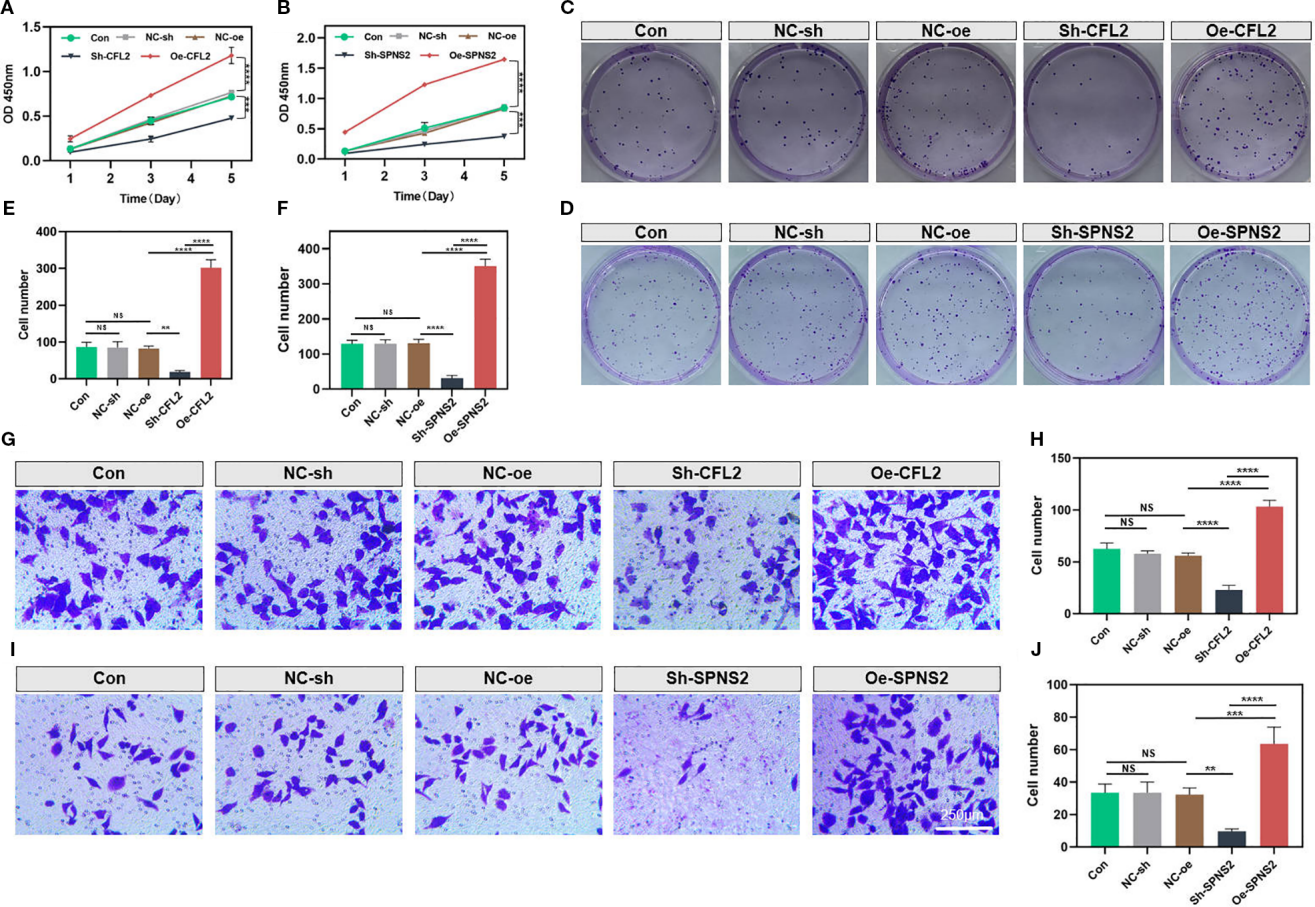
Knockdown of CFL2 or SPNS2 inhibits the proliferation and invasion of BC cells. **(A, B)** Cell proliferation was assessed using the CCK-8 assay. **(C–F)** Colony formation assay was conducted to assess the proliferation/cloning ability. **(G–J)** Transwell invasion assays were performed to evaluate the invasion capabilities of the cells. **P < 0.01, ***P < 0.001, ****P < 0.0001, ns: not significant.

CFL2 and SPNS2 showed significantly increased expression in the MDA-MB-231 cell line. Therefore, we selected MDA-MB-231 cells for knockdown experiments. Compared with other groups, the CCK-8 assay demonstrated that knockdown of CFL2 or SPNS2 significantly impaired the proliferative capacity of cancer cells (both p<0.001) (**Figures 10A, B**). The colony formation assay further revealed that the proliferation and clonogenic potential of MDA-MB-231 cells were markedly reduced after CFL2 or SPNS2 knockdown (both p<0.01) (**Figures 10C–F**). The Transwell invasion assay provided additional evidence that knockdown of CFL2 or SPNS2 significantly decreased the number of invasive cells (all p<0.01) (**Figures 10G–J**). Collectively, our findings suggest that silencing CFL2 or SPNS2 can suppress the proliferation and invasion of BC cells.

## 4 Discussion

In the construction of prognostic gene signatures, the consideration of transcriptomic ITH is crucial. Relying solely on localized features from a single biopsy sample can lead to misjudgment of prognostic risk due to sampling bias. The heterogeneity in driver gene expression profiles, such as spatial variations in ER/PR/HER2 status, caused by ITH can reduce the generalizability of prognostic models in independent cohorts. Additionally, ITH often involves the dynamic evolution of subclones associated with drug resistance pathways, such as apoptosis inhibition and immune evasion. Ignoring these features diminishes the model’s predictive power for treatment resistance. Therefore, integrating the dynamic characteristics of ITH is essential for improving the clinical applicability of prognostic models.

BC, as a highly heterogeneous tumor, exhibits diverse molecular features and biological behaviors across different regions. The TNM staging system primarily relies on anatomical parameters and fails to integrate tumor heterogeneity or immune microenvironment features. Even among patients with identical TNM stages, varying degrees of T cell infiltration in the immune microenvironment may lead to significantly divergent treatment responses and prognoses. Additionally, while PAM50 subtype uses gene expression profiling to focus on tumor-intrinsic biological traits, it omits microenvironmental characteristics like immune cell infiltration and immunosuppressive factors, limiting its predictive capacity for immunotherapy efficacy. For the first time, we employed a multi-region sequencing strategy in BC, amplifying transcriptional differences between patients while minimizing insignificant intra-tumoral transcriptional fluctuations, thereby constructing a robust risk signature. In different BC cohorts, our prognostic signature demonstrates exceptional predictive stability. It effectively differentiates patients into high- and low-risk groups and maintains independent prognostic value even after accounting for traditional clinical factors. The robustness of this signature lies in our multi-region sampling approach, which minimizes the interference of ITH in prognostic assessment. To achieve comprehensive quantitative assessment of complex biological processes, we developed a nomogram by integrating the weighted contributions of individual predictors. The nomogram captures synergistic effects between three variables, demonstrating superior translational capacity in prognostic prediction.

Our signature not only accurately predicts the prognosis of BC patients but also reveals the complex characteristics of the tumor immune microenvironment. The expression landscape of immune markers within the TME of BC patients reflects the dynamic interplay between the immune clearance and immune escape phases during tumor immunoediting. The observed features in high-risk patients (increased CD8+ T-cell infiltration, enhanced APM, elevated MHC-I expression, and elevated IPS) represent adaptive responses evolved by tumor cells under intense immune surveillance. IFN-γ released by CD8^+^ T cells can directly induce tumor cells to express MHC-I APM. The upregulated MHC-I APM enhances antigen presentation efficiency, activating CD8^+^ T cells and forming a positive feedback loop. Although this exerts anti-tumor protective effects, IFN-γ via the JAK-STAT pathway ostensibly enhances immune recognition by promoting MHC-I expression, while simultaneously inducing the expression of inhibitory molecules such as PD-L1 and IDO1 in tumor cells. This creates a balanced regulatory circuit of activation-inhibition (33–35). As shown in our study, high-risk patients show upregulated expression of immune checkpoints such as PD-1, PD-L1, and CTLA-4. Moreover, the massive infiltration of CD8+ T cells depletes local glutamine, which suppresses Foxp3 stability in regulatory T cells through the mTORC1 signaling pathway (36–38), while lactate accumulation inhibits IL-10 secretion in M2 macrophages (39, 40). This metabolic pressure transiently disrupts the immunosuppressive equilibrium; however, due to sustained checkpoint molecule expression, it ultimately drives CD8+ T cells into a functionally inactivated intermediate state (41).

CFL2 and SPNS2 may participate in the malignant progression of BC through distinct molecular mechanisms. CFL2 expression is significantly upregulated in BC tissues and cells (42). As an actin-depolymerizing factor, CFL2 regulates cytoskeletal dynamics, influencing the migratory and invasive capabilities of tumor cells (43). circ_0008673 upregulates CFL2 expression by adsorbing miR-153-3p, thereby relieving its inhibitory effect on CFL2. This promotes BC cell proliferation, migration, and invasion while inhibiting apoptosis (42). SPNS2 has been shown to promote tumor proliferation and metastasis in other cancers by activating the Akt/ERK signaling pathway (44). However, direct studies on its role in BC are limited. Additionally, previous studies have also shown that CFL2 and SPNS2 regulate immune infiltration. CFL2 is a target of miR-142-3p. Inhibition of miR-142-3p upregulates CFL2L, activating the RIG-I-mediated immune defense response and enhancing the anti-tumor function of natural killer cells (45). CFL2 modulates actin dynamics to influence cytoskeletal reorganization. In natural killer cells, suppression of its homologous protein significantly impairs cell migration, indicating its role in immune cell infiltration toward inflamed or tumor sites by remodeling the cytoskeleton (46). As for SPNS2, it transports S1P from the inside to the outside of cells, triggering S1P receptor-mediated lymphocyte migration signals (47). When SPNS2 activity is inhibited, extracellular S1P levels decrease, impairing immune cells’ ability to migrate effectively to tumor sites (48).

This study holds potential clinical significance. Our signature can be employed to quantify prognostic risk stratification in patients and guide treatment decisions. This model may resolve the heterogeneity challenge by identifying immune therapy-responsive subgroups beyond conventional chemotherapy. For high-risk patients, intensified regimens incorporating ICIs are prioritized, whereas low-risk subgroups may avoid overtreatment, thus shifting the current “one-size-fits-all” approach toward precision-based dynamic management. While this investigation revealed potential sensitivity of stratified high-risk patient populations to ICIs, it must be emphasized that these findings lack validation through clinical cohort evidence. Further validation through multi-center clinical trials is needed in the future.

## 5 Conclusion

In conclusion, this study represents the first effort to construct a prognostic signature using multi-region bulk RNA sequencing in BC. It not only provides a novel tool for prognosis assessment but also opens new avenues for understanding tumor heterogeneity and developing innovative therapeutic strategies.

## Data Availability

The original contributions presented in the study are included in the article/**Supplementary Material**. Further inquiries can be directed to the corresponding authors.

## Supplementary material

Supplementary Figure 1 Association between MATH and clinicopathological features as well as survival prognosis in TCGA-BRCA patients. **(A)** Differences in MATH scores across age groups, tumor stages, treatment conditions, and treatment types. **(B)** Kaplan-Meier survival analysis shows the overall survival difference between high and low MATH groups.

Supplementary Figure 2 quanTIseq was used to quantify the absolute abundance of immune cells in the high-risk and low-risk groups. **P* < 0.05, ***P* < 0.01, ****P* < 0.001, *****P* < 0.0001, ns: not significant.
