# Supplementary figures and images for: Intra-tumor heterogeneity-resistant gene signature predicts prognosis and immune infiltration in breast cancer
Source: Front Immunol. 2025 Sep 26;16:1598858. doi: 10.3389/fimmu.2025.1598858 (PMC12511067; doi:10.3389/fimmu.2025.1598858)

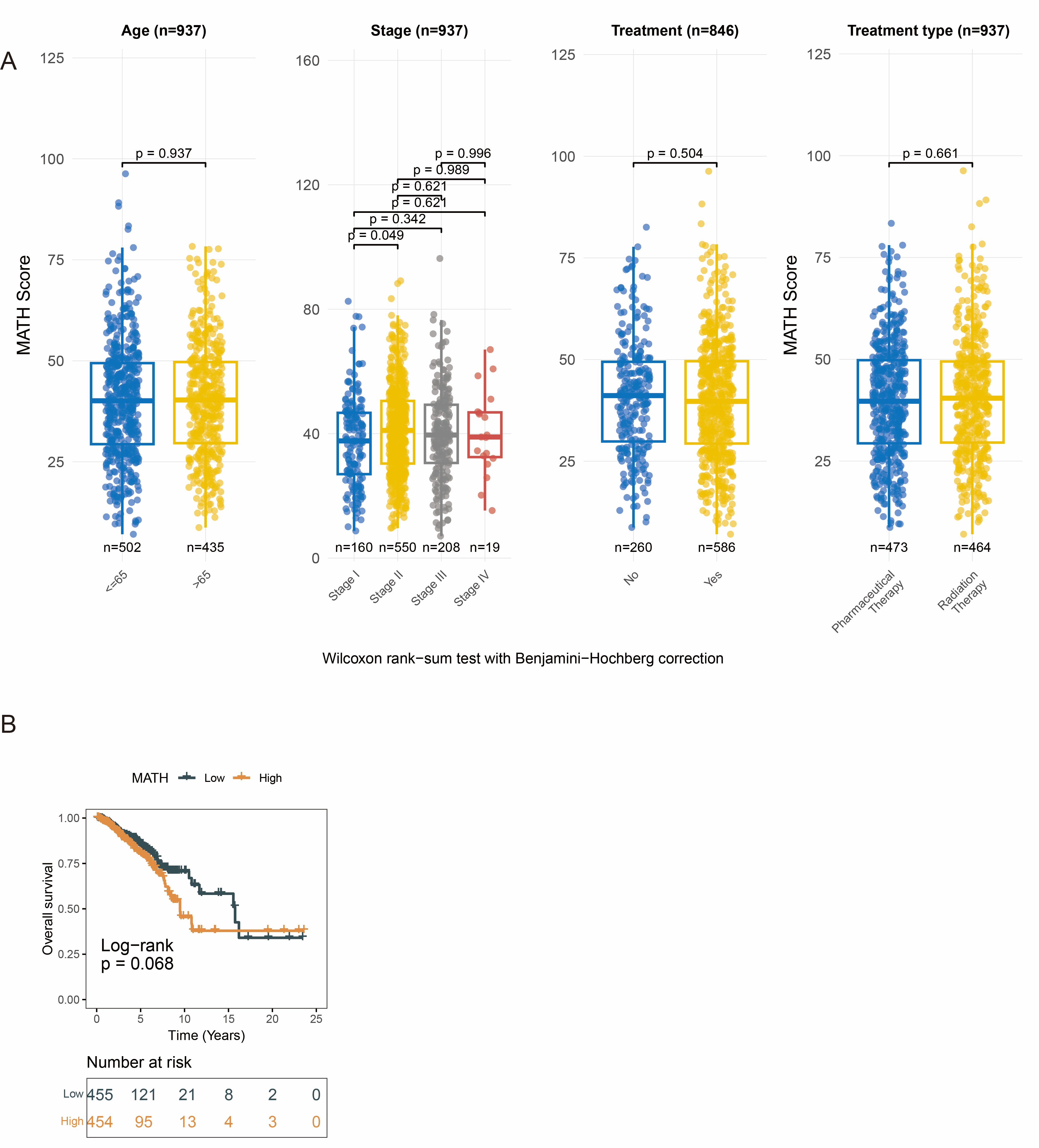

Supplement: Supplementary Figure 1 — Association between MATH and clinicopathological features as well as survival prognosis in TCGA-BRCA patients. (A) Differences in MATH scores across age groups, tumor stages, treatment conditions, and treatment types. (B) Kaplan-Meier survival analysis shows the overall survival difference between high and low MATH groups. [file Image1.jpeg]

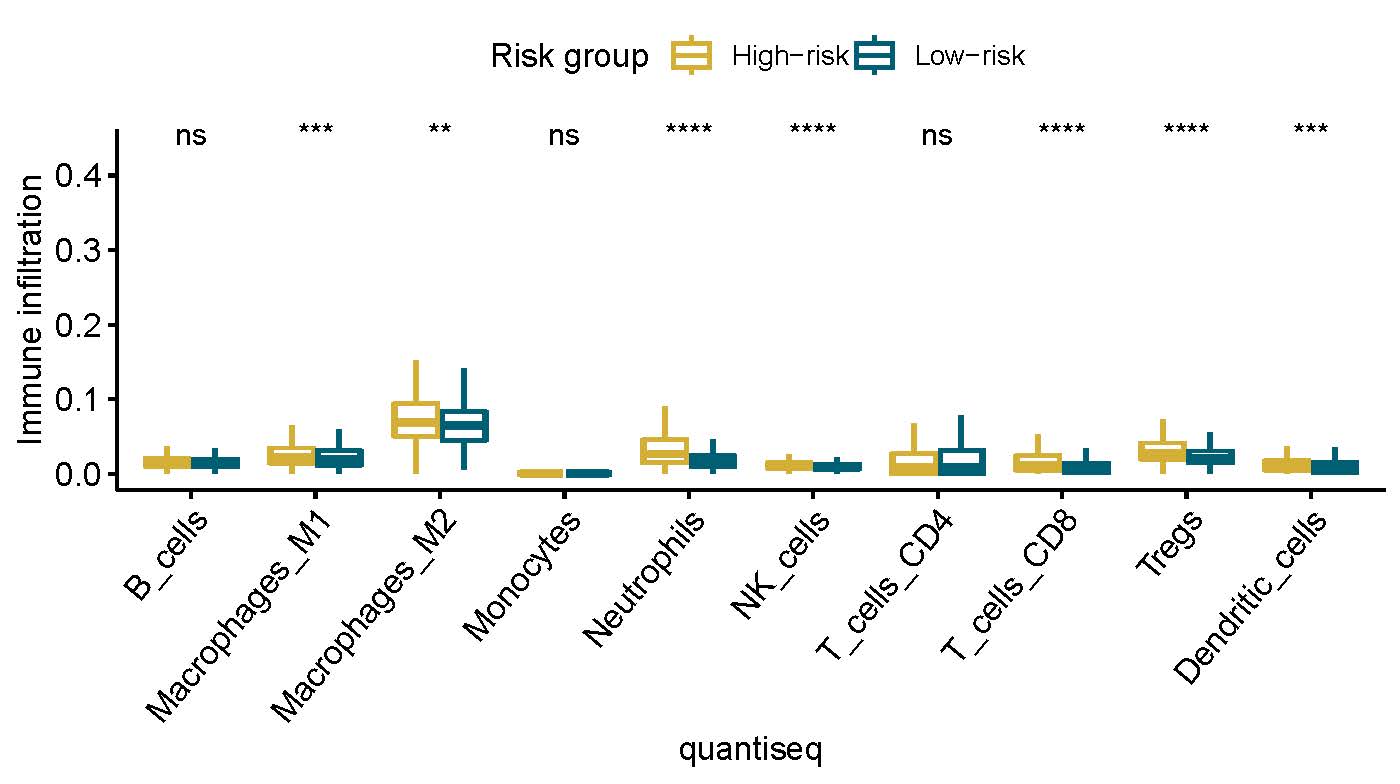

Supplement: Supplementary Figure 2 — quanTIseq was used to quantify the absolute abundance of immune cells in the high-risk and low-risk groups. *P < 0.05, **P < 0.01, ***P < 0.001, ****P < 0.0001, ns: not significant. [file Image2.jpeg]
